# Emerging trends in the coexistence of primary lung Cancer and hematologic malignancy: a comprehensive analysis of clinicopathological features and genetic abnormalities

**DOI:** 10.1186/s12935-024-03264-x

**Published:** 2024-02-24

**Authors:** Mengchen Lyu, Lifeng Luo, Ling Zhou, Xiangran Feng, Jin Yang, Ziwei Xu, Xianwen Sun, Zhiyao Bao, Xiaofei Wang, Beili Gao, Yi Xiang

**Affiliations:** 1 Department of Respiratory and Critical Care Medicine, Ruijin Hospital, Shanghai Jiao Tong University School of Medicine, Shanghai, 200000 China; 2 Institute of Respiratory Diseases, Shanghai Jiao Tong University School of Medicine, Shanghai, 200000 China; 3 Shanghai Key Laboratory of Emergency Prevention, Diagnosis, and Treatment of Respiratory Infectious Diseases, Shanghai, 200000 China; 4 Department of Respiratory Diseases, Kashgar Prefecture Second People’s Hospital, Kashi, 844000 China

**Keywords:** Primary lung cancer, Hematologic malignancy, Multiple primary cancer, Prognosis, Gene abnormality

## Abstract

**Background:**

The incidence of multiple primary cancers (MPC), especially involving primary lung cancer (PLC) and primary hematologic malignancies (PHM), is rising. This study aims to analyze clinicopathological features, gene abnormalities, and prognostic outcomes in individuals diagnosed with PLC-PHM MPC.

**Methods:**

A retrospective analysis included 89 patients diagnosed with PLC-PHM MPC at the Respiratory or Hematology Departments of Ruijin Hospital from 2003 to 2022 (a total of 842,047 people). Next-generation sequencing (NGS) assessed lung cancer specimens, while Polymerase Chain Reaction (PCR) and NGS were used for hematologic malignancy specimens. Statistical analysis involved survival analysis and Cox regression.

**Results:**

PLC-PHM MPC incidence surged from 1.67 per year (2011–2013) to 16.3 per year (2020–2022). The primary demographic for PLC-PHM MPC consists predominantly of elderly (average age 66 years) males (59.6%), with a high prevalence of metachronous MPC (89.9%). The prevailing histological types were lung adenocarcinoma (70.8%) in lung cancer (LC) and mature B-cell lymphomas (50.6%) in hematologic malignancies (HM). Notably, in a molecular testing cohort of 38 LC patients, 84.2% of lung cancer cases exhibited driver mutations, in which EGFR mutations frequence prevalent was 74.2%. In total group of 85 cases achieved a median overall survival (mOS) of 46.2 months, with a 5-year survival rate of 37.9% and advanced LC patients with LC gene mutations achieved a mOS was 52.6 months, with a 5-year OS rate of 30.6%. The median progression-free survival (PFS) following first-line treatment of 11 advanced patients with lung cancer-associated driver gene mutations is 26.6 months. Multivariate Cox regression revealed a favorable OS associated with surgery for LC, favorable PS score, adenocarcinoma pathology of LC, and the presence of genetic abnormalities associated with HM.

**Conclusion:**

PLC-PHM MPC incidence is rising, characterized by a significant proportion of lung adenocarcinoma and a high prevalence of positive driver genes, especially in EGFR. Despite suffering from two primary tumors, the PLC-PHM MPC patients had superior data of both PFS and OS, suggesting an inherently intricate background of genetic abnormalities between the two kinds of tumors.

**Supplementary Information:**

The online version contains supplementary material available at 10.1186/s12935-024-03264-x.

## Introduction

Lung cancer and hematologic malignancies are widespread forms of cancer, significantly impacting global disease burden and mortality [1]. In 2020, lung cancer incidents reached 2.21 million cases, leading to 1.8 million deaths globally. Lung cancer currently ranks as the second most frequently diagnosed malignant tumor globally and stands as the primary cause of death [2]. Hematologic malignancy (HM), on the other hand, constituted 7.5% of new cancer diagnoses and accounted for 7.8% of cancer-related deaths worldwide in 2020. The prevalence and mortality rates of HM rank it as the fifth most common cancer type [2]. The prognosis for LC and HM has consistently improved over the past decade, thanks to advancements in diagnostic techniques and the introduction of innovative therapies such as targeted therapy and immunotherapy, along with improved supportive care [3, 4]. Nonetheless, the 5-year survival rates for advanced disease are still disheartening. Moreover, exploring whether the two tumors have a worse prognosis is worthwhile.

Multiple primary cancers (MPC) refer to the occurrence of two or more synchronous or metachronous primary cancers in an individual [5]. Global MPC prevalence has risen due to aging demographics, lifestyle changes, advanced diagnostics, and increased cancer survivorship [6–8]. In 2017, an ESMO review, utilizing general population cancer registry data, indicated that 2–17% of cancer patients experienced Multiple Primary Cancers (MPC) [8]. Rising MPC trends result from increased life expectancy and cumulative cancer risk factor exposure among survivors [9].

Research indicates that individuals diagnosed with LC or HM are at a higher risk of developing secondary primary malignancies compared to the general population. In a 2019 study, it was indicated that patients with multiple myeloma had a higher likelihood of early diagnosis of breast, prostate, or lung cancer compared to the control group. Moreover, for lung cancer patients, the cancer-related mortality rate was significantly lower compared to patients with multiple myeloma (HR: 0.59; 95% CI: 0.52–0.68) [10]. A 2006 literature suggested that non-Hodgkin lymphoma patients were more prone to developing secondary lung cancer (HR: 1.31; 95% CI: 1.23–1.39) [11], and a 2007 publication reported that Hodgkin’s lymphoma patients had an increased risk of secondary lung cancer (HR: 6.7; 95% CI: 5.6–7.8) [12]. The elevated cancer risk in patients with myeloproliferative neoplasms, including an HR of 1.7 for secondary lung cancer (95% CI: 1.4–2.2) [13]. Following radiotherapy, surgically resected lung cancer patients experienced an increased risk of developing second primary solid tumors and gastrointestinal cancers, with an HR of 1.08 for secondary hematologic malignancies (95% CI, 0.84–1.37) [14]. However, current studies predominantly focus on broad cases and traditional treatment modalities, like chemotherapy and radiation. However, these cases often reflect outdated treatment approaches. Present clinical practices, especially with emerging targeted therapies for hematologic malignancies and lung cancer, exhibit a gap in comprehensive epidemiological data and clinical characteristics, necessitating further investigation.

The emergence of PLC-PHM MPC may be attributed to common environmental risk factors, direct mutational impacts of cytotoxic anticancer treatments on stem cells, and potential genetic predispositions [15–17]. Although occasional limited case reports and small retrospective series have documented the simultaneous presence of PLC and PHM in MPC [18–22], comprehensive studies investigating the epidemiological patterns, genomic profiles, prognostic determinants, and clinical outcomes of patients with PLC-PHM MPC are currently lacking on a large scale.

Consequently, we conducted a comprehensive large-sample regression cohort study on PLC-PHM MPC patients at a prominent tertiary academic hospital. Our objective was to analyze temporal trends, clinical profiles, gene status, treatment patterns, and survival outcomes of patients with PLC and PHM occurring simultaneously over the past two decades. Additionally, we aimed to identify potential risk factors associated with this cancer type by comparing the clinical outcomes of lung cancer reported in the LC literature. This study presents the most recent and thorough analysis of this rare yet increasingly recognized entity, offering insights into the unique attributes of PLC-PHM MPC patients and forming a logical foundation for customizing treatment approaches to improve their care in clinical settings.

## Methods

### Study design and participants

This retrospective observational study was conducted at Shanghai Ruijin Hospital, a leading tertiary academic medical center affiliated with Shanghai Jiao Tong University School of Medicine. We enrolled patients diagnosed with multiple primary cancers, specifically involving primary lung cancer and primary hematologic malignancies (PLC-PHM MPC), from October 2003 to October 2022, encompassing a total of 842,047 individuals. The study was approved by the Institutional Review Board of Ruijin Hospital.

The diagnosis of lung cancer was based on histopathological evidence and staged according to the 8th edition of the American Joint Committee on Cancer (AJCC) Tumor, Node, Metastasis (TNM) staging system [23]. Hematologic malignancies were classified using the fifth edition of the World Health Organization (WHO) classification [24]. MPC is defined as the occurrence of two or more primary cancers in the same individual. Synchronous MPC (sMPC) refers to cancers diagnosed within 6 months, while metachronous MPC (mMPC) indicates an interval of 6 months or more between two primary cancers [25].

Inclusion criteria were: (1) diagnosis of both primary lung cancer and primary hematologic malignancies, either synchronously or metachronously; (2) availability of complete medical records and follow-up data. Exclusion criteria included: (1) only one primary cancer; (2) hematologic malignancy or lung cancer as progression or metastasis from the first primary cancer.

### Data collection

Demographic data, clinical characteristics, pathological results, treatment approaches, and survival outcomes were extracted through a systematic review of electronic medical records. Data collection included age, sex, smoking history, cancer history, ECOG performance status, cancer stage at diagnosis, pathological type, treatment modalities, dates of diagnosis for each primary cancer, clinical outcomes, and survival status.

Genomic profiling of lung cancer specimens was conducted using Next Generation Sequencing (NGS), targeting hotspot regions in 10–68 frequently mutated genes. For hematologic malignancy specimens, gene abnormalities were characterized in 35 patients using Polymerase Chain Reaction (PCR) and NGS.

### Outcome measures

The primary outcome measure was overall survival (OS), defined as the time from the diagnosis of PLC-PHM MPC to death from any cause or the last follow-up. Survivors were censored at the last follow-up date.

### Statistical analysis

Continuous variables were presented as medians (range) and compared using the Mann-Whitney U test. Categorical variables were expressed as numbers (percentage) and compared using the Pearson Chi-square test or Fisher’s exact test as appropriate. OS was analyzed using the Kaplan-Meier method and survival curves were compared via the log-rank test. Prognostic factors for OS were identified using univariate Cox regression models. Hazard ratios (HR) and 95% confidence intervals (CI) were calculated. Factors with *P* < 0.1 in univariate analyses were entered into the multivariate Cox model using a forward stepwise method to determine independent prognostic factors. Two-sided P values < 0.05 were considered statistically significant. Statistical analysis was performed using SPSS 22.0. Pheatmap and ggplot2 (v.3.3.6, http://ggplot2.org/) were used for visualizations.

## Results

### Patient characteristics

**Fig. 1:**
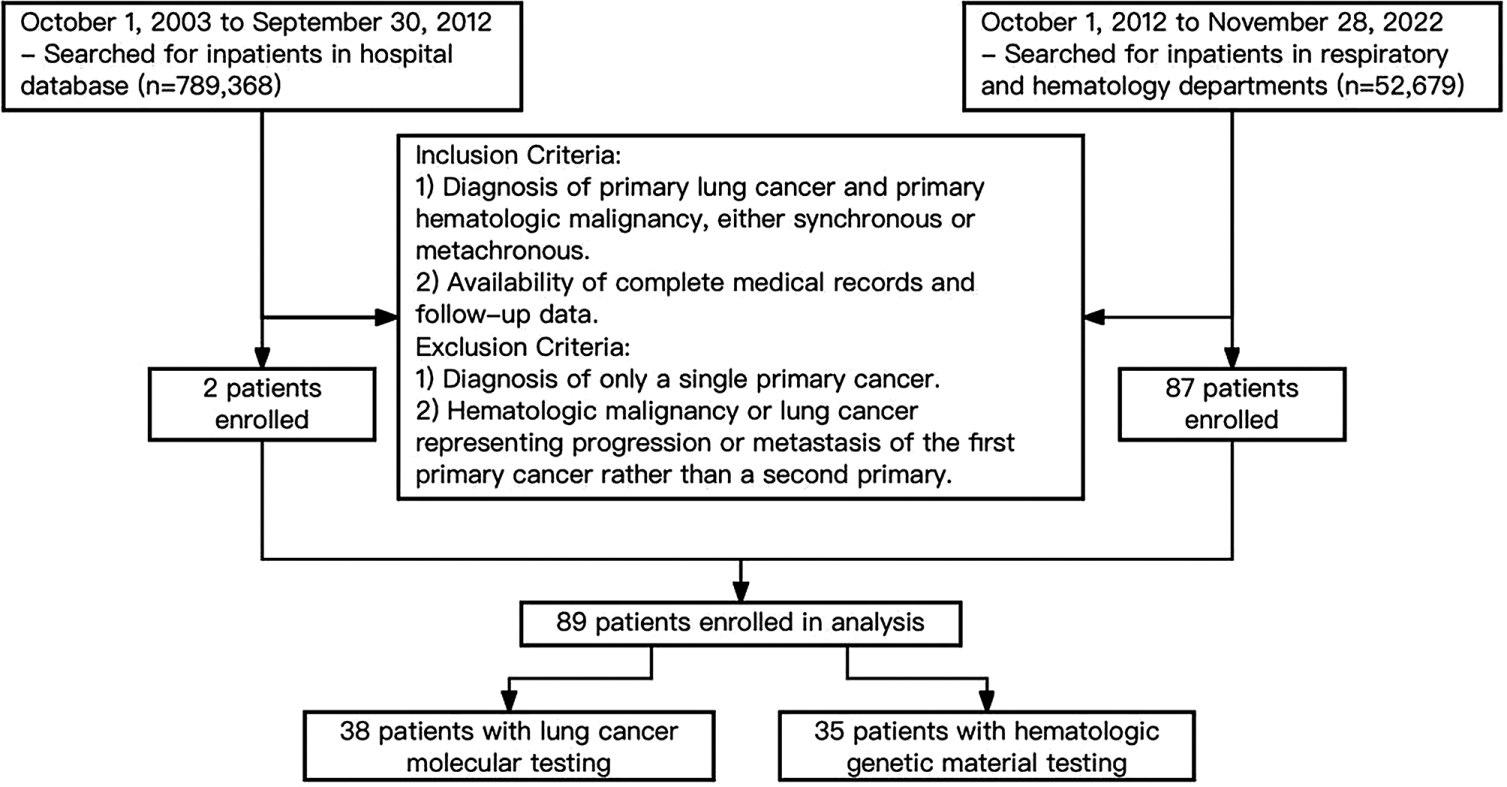
Flow chart of the study design

**Fig. 2:**
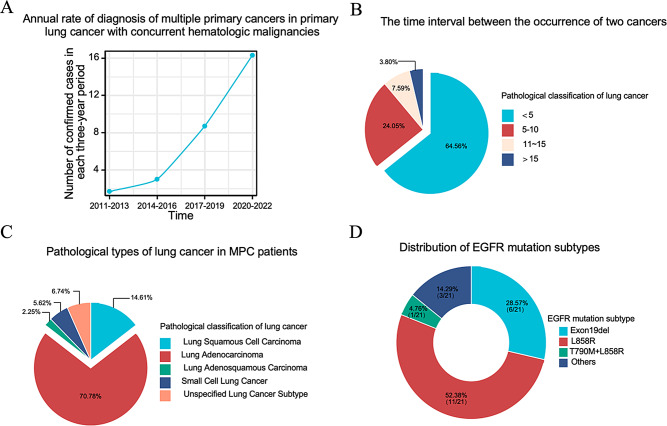
Epidemiological, clinical, and molecular characteristics of lung cancers in patients with multiple primary cancers involving lung cancer and hematologic malignancies. (**A**) Annual incidence rates of multiple primary cancers involving lung cancer and concurrent hematologic malignancies over the study period. (**B**) Time intervals between diagnosis of lung cancer and hematologic malignancy. (**C**) Distribution of lung cancer histological subtypes. (**D**) Frequencies of EGFR mutation subtypes identified among EGFR-mutant lung cancers

In this retrospective analysis of 89 patients with PLC and HM MPC (Fig. 1), the incidence has surged from 1.67 per year (2011–2013) to 16.3 per year (2020–2022) (Fig. 2A).

The median age at diagnosis was 66 years (range: 38–90 years), with 23.6% under 60 and 76.4% aged 60 or older. The male-to-female ratio was 1.47:1, with 31.5% having a smoking history (exclusively male smokers) and 10.1% reporting a family history of cancer. Comorbidities were present in 38.2% of patients, primarily hypertension. The majority had two primary cancers (89.9%), while a smaller subset had three or more (10.1%) (Table 1).

**Table 1:** General clinical characteristics of MPC patients

Clinical Feature		Number (*n* = 89)	Percentage
Gender	Male	53	59.56%
	Female	36	40.44%
Age at Diagnosis	< 60 years	21	23.60%
	≥ 60 years	68	76.40%
Smoking History	Yes	28	31.46%
	Never	61	68.54%
Family History	Yes	9	10.11%
	No	80	89.89%
History of Comorbidities	Yes	34	38.20%
	No	55	61.80%
Number of Primary Cancers	Dual Primary Cancers	80	89.89%
	Triple Primary Cancers or more	9	10.11%

### Interval between lung cancer and hematologic malignancy

In the cohort of 89 MPC cases, 10 were synchronous, and the remaining 79 were metachronous. For metachronous cases, the median duration between LC and HM diagnosis was 42.8 months (range: 6.8-221.3 months), with 64.6% occurring within a 5-year timeframe (Fig. 2B).

### Pathology of cases with triple or more primaries

In the subset of 9 patients presenting with three or more primary cancers, the observed cancer types exhibited notable heterogeneity, as outlined in Table 2. The majority of hematologic malignancies were identified as multiple myeloma (*n* = 4) and lymphoma (*n* = 3). In the cases of lung cancer, adenocarcinomas predominated, except for a singular instance of squamous cell carcinoma.

**Table 2:** Distribution of Third or More Primary Cancers in Patients with Primary Lung Cancer Complicated by Hematological Malignancies

ID	Gender	First Primary Cancer	Second Primary Cancer	Third Primary Cancer	Fourth Primary Cancer
38	Female	Breast Cancer	Lung Adenocarcinoma	B-Cell Lymphoma	
42	Male	Lung Adenocarcinoma	Renal Clear Cell Carcinoma	Gastric Stromal Tumor	T-Cell Lymphoma
43	Female	Breast Cancer	Colorectal Cancer	B-Cell Lymphoma	Lung Adenocarcinoma
45	Male	B-Cell Lymphoma	Bladder Cancer	Lung Adenocarcinoma	
46	Female	Cervical Cancer	Lung Squamous Cell Carcinoma	Acute Leukemia	
47	Female	Breast Cancer	Multiple Myeloma	Lung Adenocarcinoma	
65	Male	Stomach Cancer	Multiple Myeloma	Lung Adenocarcinoma	
73	Female	Thyroid Cancer	Lung Adenocarcinoma	Multiple Myeloma	
81	Male	Multiple Myeloma	Renal Clear Cell Carcinoma	Lung Adenocarcinoma	

### Characteristics of lung cancer

The median age at lung cancer diagnosis was 65 years, with non-small cell lung cancer constituting 87.6% of cases. Within these cases, lung adenocarcinoma was the most prevalent subtype at 70.8% (Fig. 2C). At diagnosis, 43.8% of patients were stage I, 5.6% were stage II, 13.5% were stage III, and 18% were stage IV. The majority demonstrated a good performance status (PS), with scores of 0–1 in 76.4% and scores of ≥ 2 in 4.5%. Among the 89 MPC patients, 66.3% had a prior HM diagnosis, while 33.7% had LC as their initial malignancy (Table 3).

**Table 3:** Clinical Characteristics of Lung Cancer Patients with MPC

Clinical Feature		Cases	Proportion (%)	Clinical Feature		Cases	Proportion (%)
Age	< 60 years	26	29.21%	PS Score	0–1	68	76.40%
	≥ 60 years	63	60.79%		≥ 2	4	4.50%
Pathological Type	Squamous	13	14.61%		Unclear	17	19.10%
	Adenocarcinoma	63	70.79%	Surgical History	Yes	59	66.29%
	Adenosquamous	2	2.25%		No	26	25.84%
	Small Cell	5	5.62%		Unclear	4	7.87%
	NSCLC-NOS	6	6.74%	Molecular Abnormality	Yes	32	35.96%
Clinical Stage	Stage I	39	43.82%		No	6	6.74%
	Stage II	5	5.62%		Unclear	51	57.30%
	Stage III	12	13.48%	Hematological Malignancy Occurred First	Yes	59	66.29%
	Stage IV	16	17.98%	Lung Cancer Occurred First	Yes	30	33.70%
	Unclear	17	19.10%				

*Note* NSCLC-NOS refers to Non-Small Cell Lung Cancer-Not Otherwise Specified

### Molecular profile of lung cancer

**Fig. 3:**
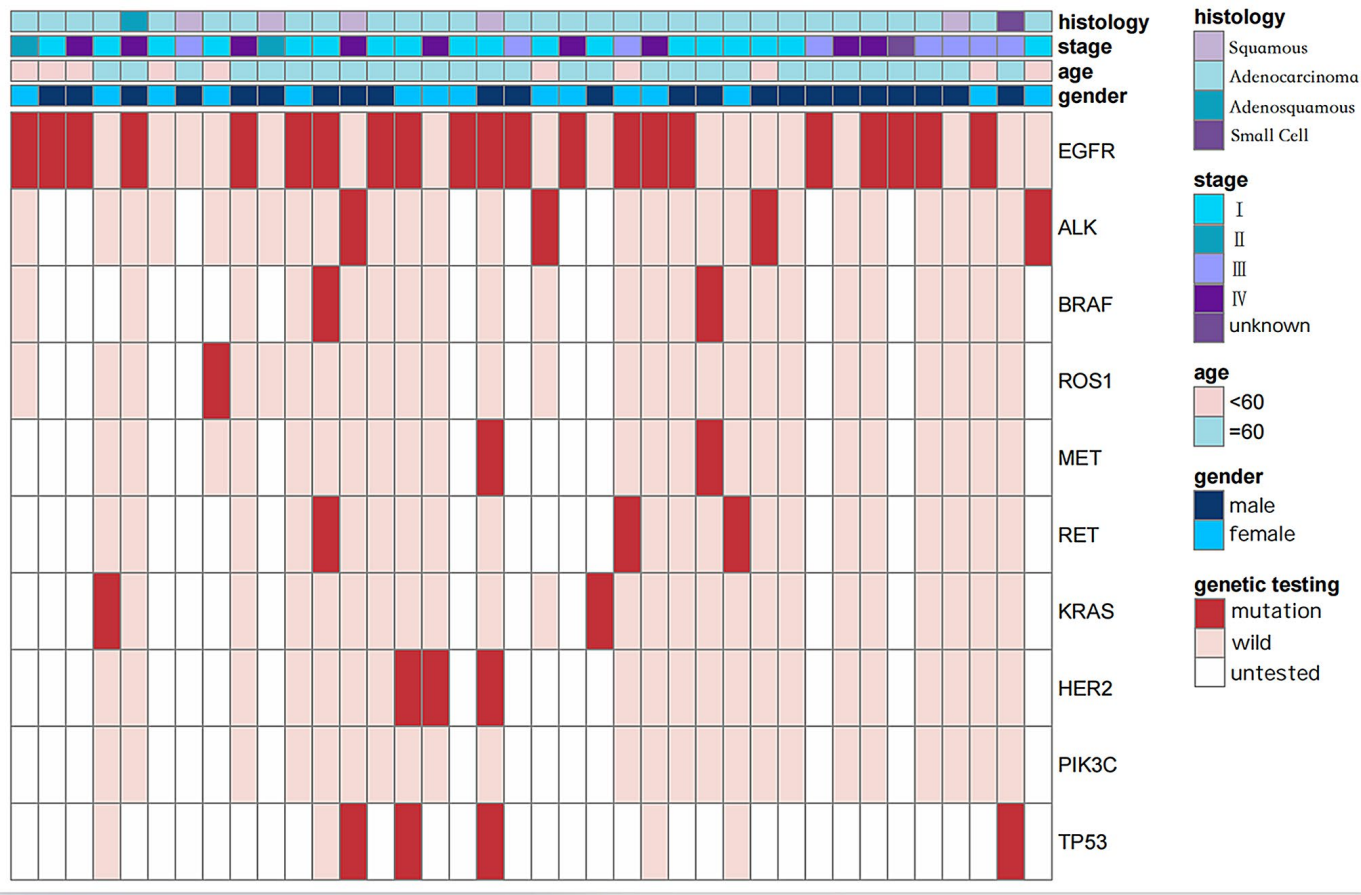
Ten lung cancer driver gene mutation states in 38 patients

In a cohort of 38 LC patients, molecular testing revealed driver gene mutations in 84.2% of cases. The most prevalent driver mutations and their frequencies were as follows: EGFR at 74.2%, TP53 at 50%, ALK at 13.8%, RET at 13%, HER2 at 13%, MET at 8.3%, and BRAF at 8% (Supplementary Table 1 and Fig. 3). Among the EGFR mutations, the most frequent was the EGFR-L858R mutation at 55.26% (11/21), followed by the EGFR-19del mutation at 32% (6/21) (Fig. 2D).

### Characteristics of hematologic malignancies

The median age of diagnosis for hematologic malignancies was 64 years. The most prevalent subtype was mature B-cell lymphoma, accounting for 50.6% of cases, followed by acute myeloid leukemia (14.6%), NK/T cell lymphoma (11.2%), plasma cell neoplasms (10.1%), myelodysplastic syndromes (4.5%), and Hodgkin lymphoma (3.4%). Genetic abnormalities were assessed in 35 patients, with a detection rate of 91.4%. These abnormalities included chromosomal aberrations (63.6%), gene rearrangements (69.6%), and gene mutations (86.9%) (Table 4).

**Table 4:** Clinical Characteristics of Patients with Hematologic Malignancies

Clinical Feature		Number of Cases	Proportion (%)
Age	< 60 years	31	34.83%
	≥ 60 years	58	65.17%
Pathology	Mature B-cell Lymphoma	45	50.56%
	Acute Myeloid Leukemia	13	14.61%
	NK/T-cell Lymphoma	10	11.24%
	Non-Hodgkin Lymphoma	5	5.62%
	Myelodysplastic Syndrome	4	4.49%
	Hodgkin Lymphoma	3	3.37%
Genetic Material Alteration	Present	32	35.96%
	Absent	3	3.37%
	Unknown	54	60.67%
Chromosomal Aberrations	Present	14	63.64%
	Absent	8	36.36%
Gene Rearrangements	Present	16	69.57%
	Absent	7	30.43%
Gene Mutations	Present	20	86.96%
	Absent	3	13.04%

### Treatments administered for lung cancer and hematologic malignancies

In the cohort of 89 MPC patients, 66.3% (59/89) had undergone previous surgical intervention for lung cancer. For other patients, targeted therapy was administered in 19.1% (17/89), immunotherapy in 3.4% (3/89), chemotherapy in 5.6% (5/89), and radiotherapy in 2.2% (2/89). The treatment details for lung cancer were unknown in 3.4% (3/89) of cases.

Regarding the treatment of hematological tumors, targeted therapy accounted for 7.9% (7/89), immune-related therapy for 6.7% (6/89), and chemotherapy for 67.4% (60/89). The detailed treatment methods are provided in Supplementary Table 2.

### Survival outcomes

**Fig. 4:**
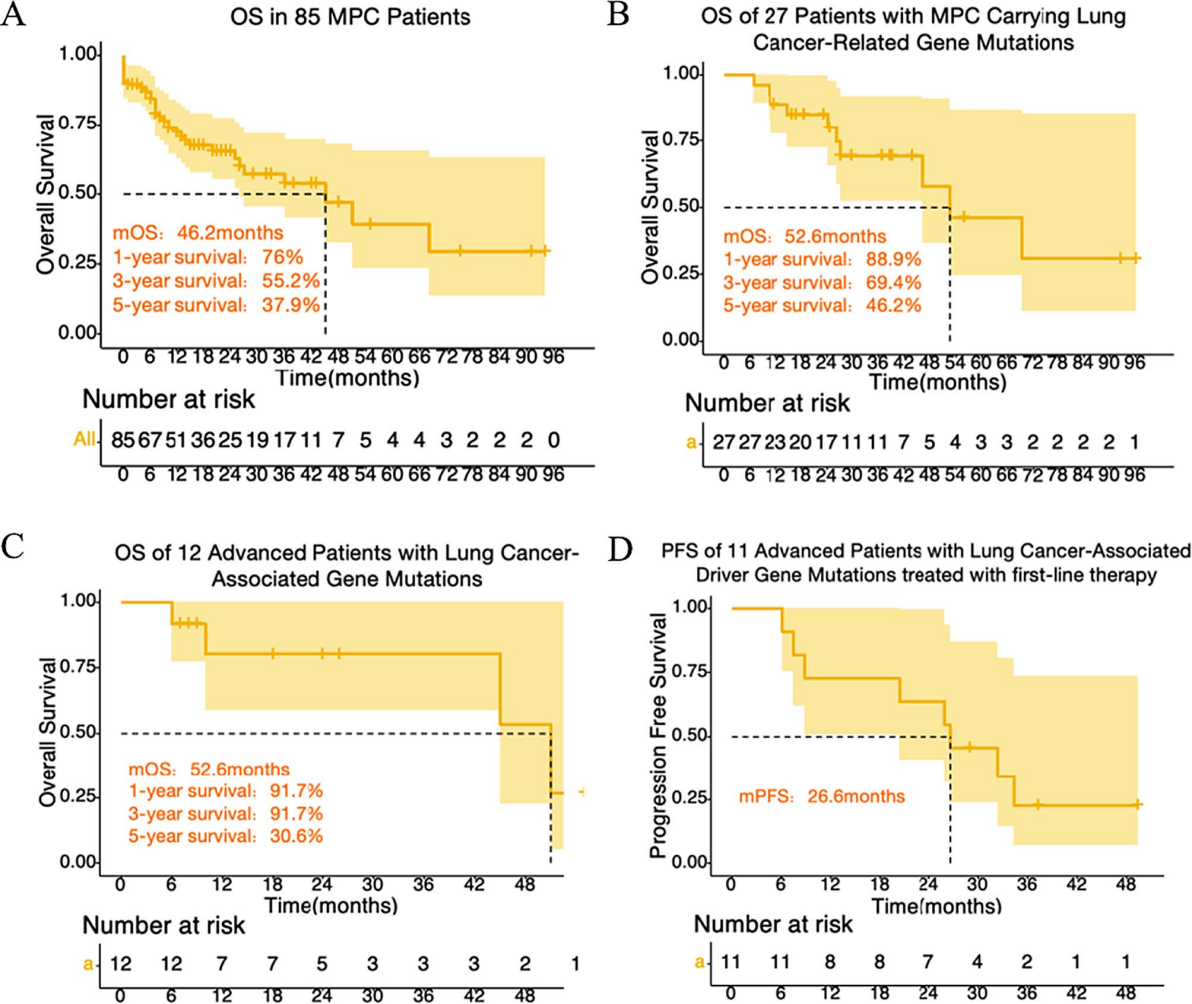
Overall survival (OS) and Progression-free survival (PFS). (**A**) OS in 85 MPC patients. (**B**) OS in 27 patients with MPC carrying lung cancer-related gene mutations. (**C**) OS in 12 advanced patients with lung cancer-associated driver gene mutations. (**D**) PFS in 11 advanced patients with lung cancer-associated driver gene mutations treated with first-line therapy. A total of 12 patients with advanced lung cancer gene mutations were enrolled, one of whom with an unknown treatment regimen was excluded from PFS analysis

In the cohort of 85 MPC patients analyzed for survival, the median overall survival (mOS) was 46.2 months (95% CI 21.9–70.6). The 3- and 5-year OS rates were 55.2% and 37.9%, respectively (Fig. 4A). Among the 38 patients undergoing LC molecular testing, the mOS was 52.6 months in 27 cases with positive mutations, displaying 3- and 5-year OS rates of 69.4% and 46.2%, respectively (Fig. 4B). For the 12 advanced LC patients with LC gene mutations, the mOS was 52.6 months (95% CI: 42.3–62.8), with 3- and 5-year OS rates of 91.7% and 30.6%, respectively (Fig. 4C). The median progression-free survival (mPFS) following first-line treatment of 11 advanced patients with lung cancer-associated driver gene mutations is 26.6 months (Fig. 4D).

**Fig. 5:**
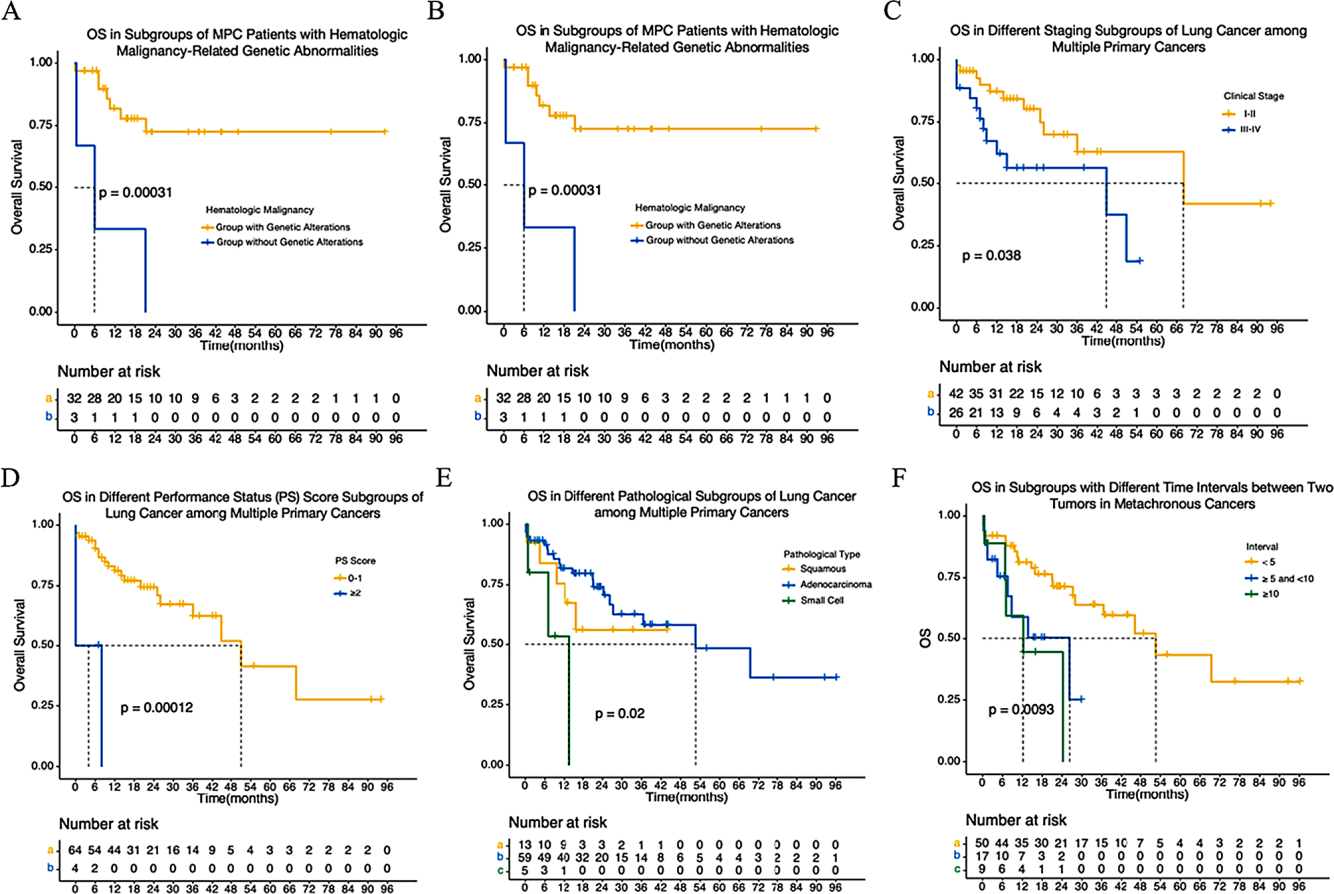
The survival outcomes of each subgroup. (**A**) OS in hematologic malignancy-related genetic abnormalities. (**B**) OS in lung cancer surgical history. (**C**) OS in staging subgroups of lung cancer among multiple primary cancers. (**D**) OS in PS score of lung cancer. (**E**) OS in pathological subgroups of lung cancer. (**F**) OS in subgroups with different time intervals between two tumors

Comparatively, MPC patients with genetic abnormalities related to HM had more favorable outcomes, evident in a 5-year overall survival rate of 72.2% compared to 19.5% in wildtype cases (*P* < 0.001) (Fig. 5A). Those with a history of LC surgery showed a significantly longer mOS of 69.4 months (95% CI: 5.7–133, *P* < 0.001) than patients without such a history (Fig. 5B). Additionally, patients with stage I-II LC had a significantly longer mOS of up to 69.4 months (95% CI: 12.1-126.6, *P* < 0.05) **(**Fig. 5C). LC patients with a PS score of 0–1 exhibited a significantly longer mOS of 52.6 months (95% CI: 32.5–72.7, *P* < 0.001) (Fig. 5D). Furthermore, patients diagnosed with adenocarcinoma experienced a prolonged mOS of 69.1 months (95% CI: 18.8–86.4, *P* < 0.05) (Fig. 5E). Among the 75 patients with metachronous MPC, those with an interval of occurrence between LC and HM within 5 years demonstrated a significantly longer mOS of 51 months (95% CI: 30.0, 72.0, *P* < 0.05) (Fig. 5F).

**Fig. 6:**
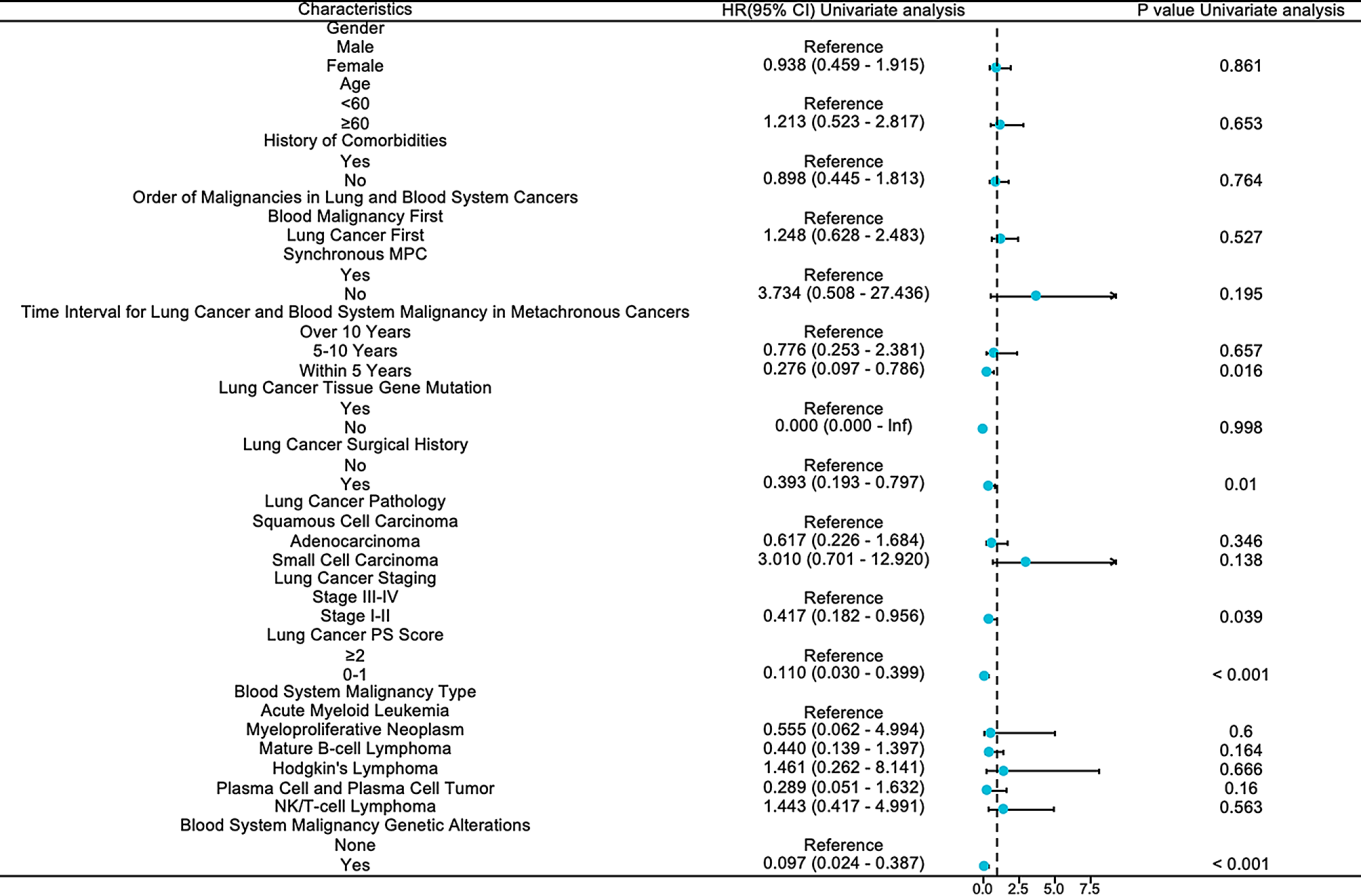
Univariate analysis for overall survival

In the univariate analysis, several factors were significantly associated with OS, including a history of surgery for LC (HR: 0.393, 95% CI: 0.193–0.797, *P* < 0.05), early stage of LC (HR: 0.417, 95% CI: 0.182–0.956, *P* < 0.001), favorable PS score (HR: 0.110, 95% CI: 0.030–0.399, *P* < 0.05), a cancer interval within 5 years (HR: 0.276, 95% CI: 0.097–0.786, *P* < 0.05), and the presence of genetic abnormalities associated with HM (HR: 0.097, 95% CI: 0.024–0.387, *P* < 0.001) (Fig. 6 and Supplementary Table 3).

**Fig. 7:**
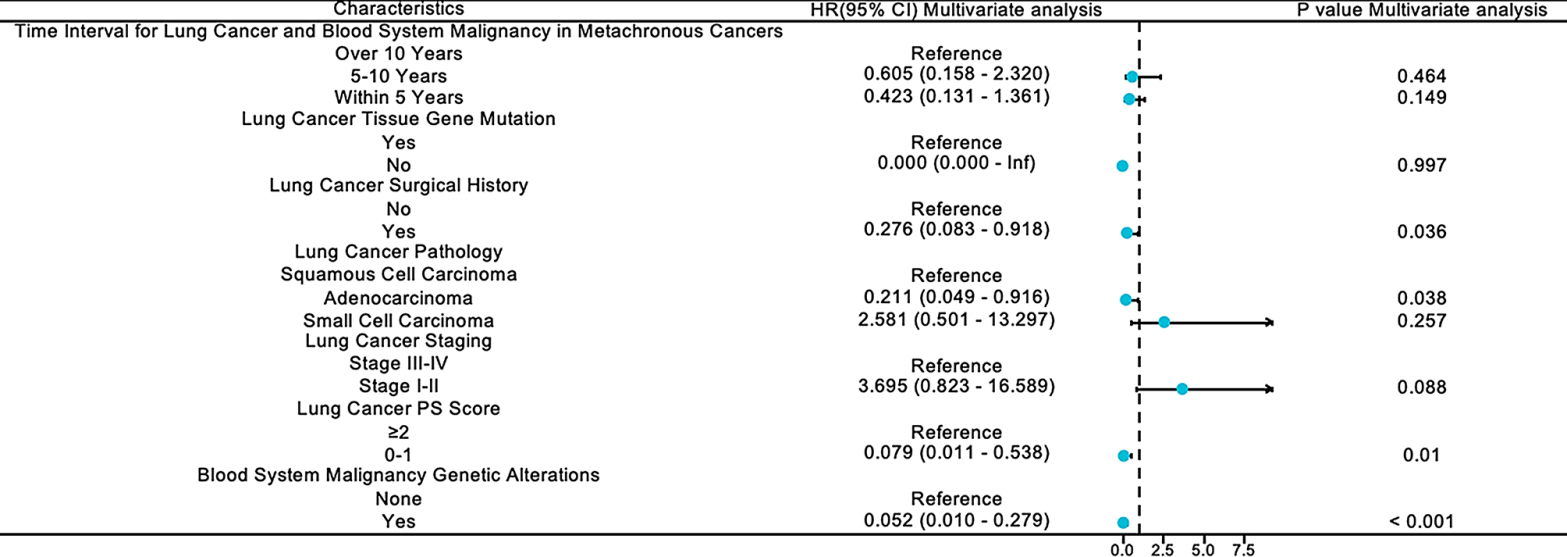
Multivariate analysis for overall survival

The subsequent multivariate Cox regression analysis revealed independent prognostic factors, including a history of surgery for LC (HR: 0.276, 95% CI: 0.083–0.918, *P* < 0.05), a favorable PS score (HR: 0.079, 95% CI: 0.011–0.538, *P* < 0.05), adenocarcinoma pathology of LC (HR: 0.211, CI: 0.049–0.916, *P* < 0.05), and the presence of genetic abnormalities associated with HM (HR: 0.052, 95% CI: 0.010–0.279, *P* < 0.001) (Fig. 7 and Supplementary Table 3).

### Durable response to RET inhibition in a patient with RET fusion-positive lung adenocarcinoma and chronic lymphocytic leukemia

**Fig. 8:**
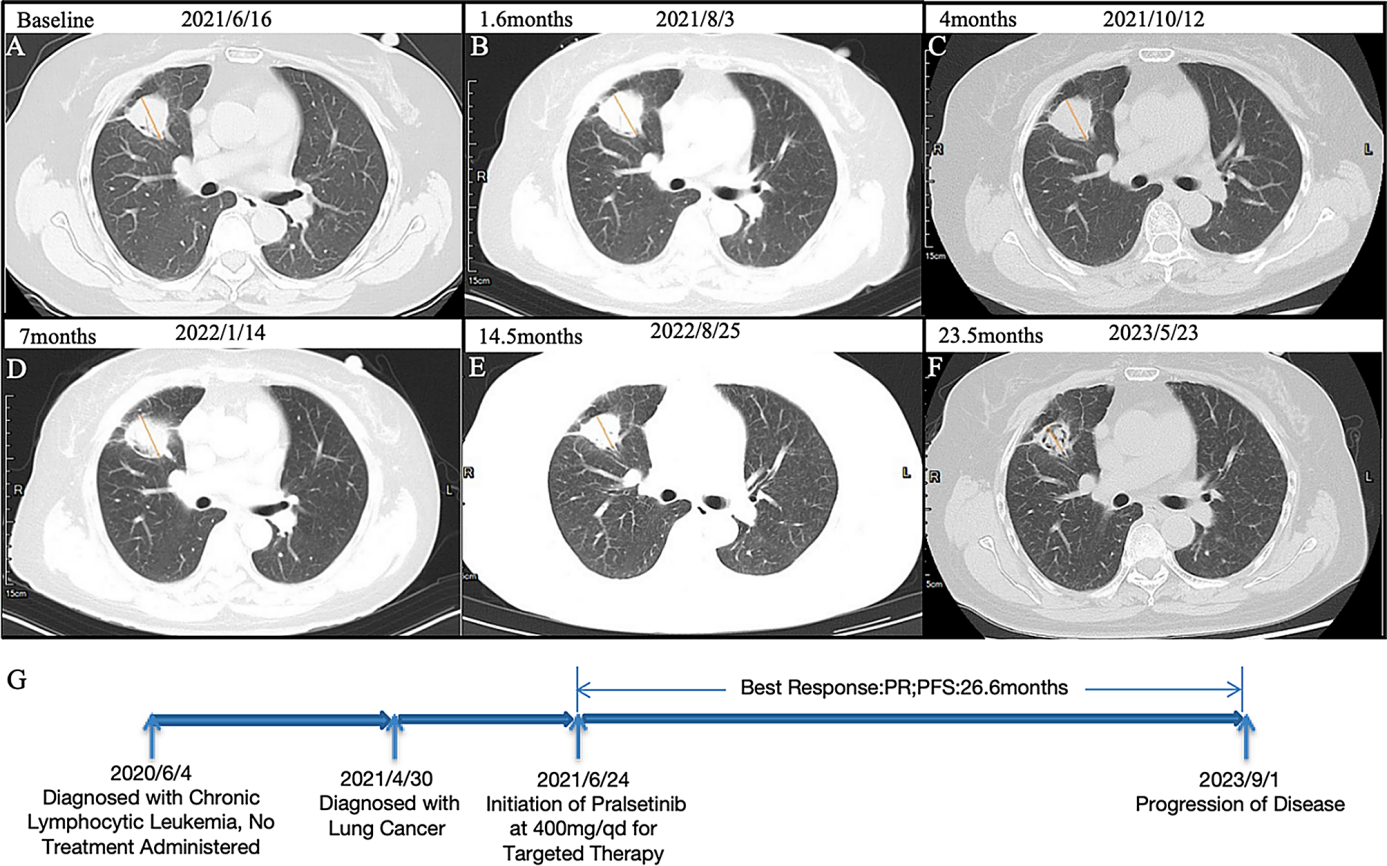
Chest CT scans and Treatment Timeline in the Case. (**A**) Baseline scan before treatment; (**B**) Scan after 1.6 months of treatment; (**C**) Scan after 4 months of treatment; (**D**) Scan after 7 months of treatment; (**E**) Scan after 14.5 months of treatment; (**F**) Scan after 23.5 months of treatment; (**G**) Timeline illustrating the course of treatment

**Fig. 9:**
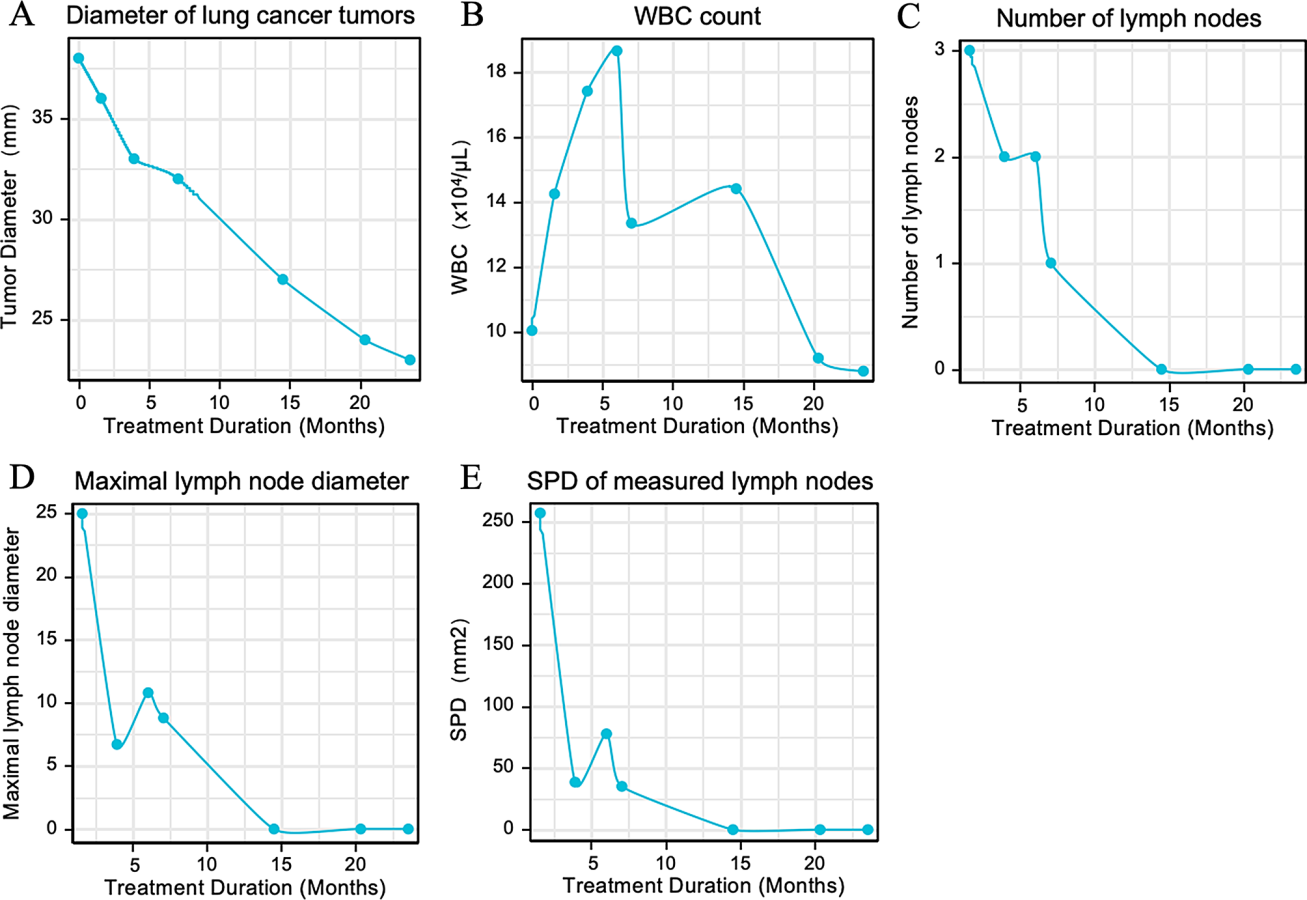
Assessment of lung cancer and lymph node characteristics during treatment of the case. (**A**) Changes in lung cancer tumor diameter. (**B**) White blood cell (WBC) count dynamics. (**C**) Lymph node count variation. (**D**) Maximal lymph node diameter changes. (**E**) SPD of measured lymph nodes, SPD represents the sum of the products of the perpendicular diameters of measured lymph nodes

In June 2020, a 59-year-old female patient presented with chronic lymphocytic leukemia, followed by a diagnosis of lung adenocarcinoma in April 2021. Molecular profiling of the lung tumor revealed an oncogenic RET fusion. Treatment with the RET inhibitor pralsetinib commenced on June 16, 2021, with no intervention for hematological tumors. The Progress Free Survival (PFS) was 26.6 months. Administration of the RET inhibitor pralsetinib resulted in significant responses in both malignancies, achieving a partial response in the lung lesions and effective responses in hematologic tumors based on chest CT, lymph nodes, and WBC findings (Figs. 8 and 9).

## Discussion

Recently, studies conducted in 2022 and 2023 using SEER database patient information (spanning from 1973 to 2015 and 1975–2018, respectively) have implicated Hodgkin’s lymphoma in the development of subsequent lung cancer, associating this relationship with radiotherapy. The 2022 study identified an increased risk of secondary lung cancer related to radiation therapy (95% CI: 1.002–1.55) [26], while the 2023 study established a correlation between primary lung cancer and subsequent tumors with radiation therapy (95% CI: 1.08–1.35) [14]. Notably, these studies overlooked the pathological and genetic characteristics of lung cancer.

Over the last decade, targeted therapies for lung cancer and recent advancements in blood-based targeted treatments have gained significant progresses. Given the current lack of research on the relationship between novel treatment modalities, genetic features, and prognosis in PLC-PHM MPC, our study aims to address this gap by focusing on epidemiology, clinical information, and genetic characteristics, contributing to a more comprehensive understanding of this complex interplay.

In this comprehensive 20-year retrospective study, we systematically analyzed 89 patients diagnosed with MPC involving PLC and PHM at a tertiary academic hospital. Our findings revealed a significant increase in PLC-PHM MPC incidence, with an annual trend showing nearly a 9-fold rise from 2011 to 2022. These results align with previous extensive analyses of population-based cancer registry data [6–8]. The heightened risk of secondary primary malignancies and associated risks among individuals with cancer can be attributed to factors such as advancements in tumor diagnosis, promotion of genetic testing, improvements in treatment efficacy, and extended patient survival [7, 10, 27, 28]. It is crucial to note that treatment itself may induce gene mutations, enhancing the potential for subsequent cancer development [9, 15]. Consequently, research underscores the imperative of personalized treatment approaches and a comprehensive evaluation of the potential risks of future MPC.

Smoking is a significant risk factor for numerous primary cancers, with smokers having a hazard ratio for multiple primary cancers 1.3-fold greater than non-smokers [29]. However, our findings deviate from this established association. Only 31.46% (28 out of 89) of MPC patients had a history of smoking, which may be attributed to the 1.47:1 male-to-female ratio in our study and a 52.8% smoking ratio in male patients. Our cohort predominantly consisted of elderly males, aligning with previous studies [1, 2, 30, 31]. In our male patient population, the smoking rate was 52.8%. However, only 31.46% of the overall group were smokers. Considering the biases related to gender and age, this discrepancy does not conclusively indicate a relationship between smoking and the occurrence of MPC.

In this study, 10.11% of patients exhibited familial tumor predisposition, consistent with previous investigations [32]. Additionally, 38.2% had underlying diseases. Our findings align with prior studies, underscoring the need for vigilance in individuals diagnosed with MPC, especially those with comorbidities such as hypertension, diabetes, or chronic inflammation, regarding the potential development of secondary primary tumors [33–35].

The majority of cases in our study (89.9%) were characterized by metachronous presentation, consistent with previous retrospective analyses reporting a higher incidence of metachronous diagnoses in cases of multiple primary cancers [36, 37]. Furthermore, our study revealed that a shorter interval (within 5 years) between the diagnosis of PLC and PHM was associated with a more favorable prognosis, aglining with results of several studies on head-and-neck and LC MPC [38, 39]. However, further validation is needed, as other investigations had not observed significant differences in survival based on synchronous versus metachronous presentation [36, 37].

In this study, the prevalence of lung adenocarcinoma histology was significantly higher (70.8%) compared to the general PLC population (55%), indicating increased susceptibility to MPC among patients with adenocarcinoma. Regarding hematologic malignancies, mature B-cell lymphoma (50.56%) was the most prevalent subtype, followed by acute myeloid leukemia (14.61%), showing a higher distribution compared to epidemiological data (40% and 10% respectively) in HM [1].

Furthermore, the driver mutation rate in PLC within our PLC-PHM MPC cohort was notably elevated (84.2%), with the EGFR mutation rate reaching 74.19%, surpassing rates observed in previous LC cohorts (32.2%) [40–43]. The high mutation burden suggests that genetic susceptibilities, particularly EGFR mutations, may play a role in the development of PLC-PHM MPC tumors. Additional genomic profiling studies are required to ascertain whether patients with PLC-PHM MPC also exhibit unique genomic abnormalities contributing to the development of MPC.

Previous studies have shown a 5-year lung cancer survival rate of 19.8% [44]. Among these, advanced-stage EGFR-positive lung cancer patients typically have a mOS of 45.7 months [44]. Our research indicates that our cohort’s survival rates surpass these figures. The mOS for patients with PLC-PHM MPC was 46.2 months, with a 5-year survival rate of 37.9%. Advanced LC patients with gene mutations had an even higher mOS of 52.6 months and a 5-year OS rate of 30.6%. The mPFS following first-line treatment of 11 advanced patients with lung cancer-associated driver gene mutations is 26.6 months, outperforming reported outcomes of three third-generation EGFR-TKIs in the treatment of advanced LC (mPFS: 18.9–20.8 months) [45–47]. This survival advantage can be partially attributed to the elevated mutation burden, enabling personalized targeted therapy [48–52].

In addition, we observed clinical factors associated with the prognosis of PLC-PHM MPC. These factors include the time interval between the occurrence of two primary cancers, genetic findings related to hematologic malignancies, as well as the stage and performance status of LC. Our MPC cohort exhibited a higher prevalence of oncogenic alterations, according with a longer OS, compared to cohorts of solitary lung cancers [40, 50, 53, 54]. However, our statistical analysis did not establish a correlation between lung cancer driver gene mutations and survival prognosis. This may be attributed to the limited size of our data sample and the substantial variability in clinical outcomes among patients with different gene mutations. Additionally, the inadequacy of our sample size, coupled with missing data, precluded the possibility of conducting subgroup analyses. It is plausible that targeted therapy guided by genomic profiling was administered in many cases.

Several limitations should be acknowledged in this study. Firstly, being conducted at a single tertiary academic hospital with a modest sample size, findings may lack generalizability. Large-scale multi-institutional collaborations are recommended for validation and expansion. Secondly, retrospective design limits data capture, particularly on systemic therapy specifics impacting survival outcomes. Thirdly, further germline and somatic genomic analyses would enhance the understanding of genetic predispositions in MPC. Lastly, despite two-decade patient monitoring, OS data may be incomplete due to the extended lifespan observed in current cohorts. Continued follow-up is warranted to reveal additional secondary malignancies and OS events.

## Conclusion

The primary observations can be briefly summarized as follows:

- The incidence of PLC-PHM MPC showed an increasing annual trend over the past two decades.
- PLC-PHM MPC patients were predominantly elderly males and non-smokers. Metachronous MPC was more common.
- Lung adenocarcinoma and mature B-cell lymphoma were the most frequent cancer types.
- A high frequency of targetable driver mutations, such as EGFR, was observed in lung cancer specimens.
- The median overall survival of PLC-PHM MPC patients reached 46.2 months, with a corresponding 5-year survival rate of 37.9%. For the 12 advanced LC patients with LC gene mutations, the mOS was 52.6 months, with 5-year OS rates of 30.6%. The mPFS following first-line treatment of 11 advanced patients with lung cancer-associated driver gene mutations is 26.6 months. Survival outcomes appeared better compared to historical cohorts of solitary primary lung cancer.
- Shorter interval between two cancers, positive hematologic malignancy genetic findings, history of lung cancer surgery, early TNM stage, pathology of lung adenocarcinoma, and better performance status were associated with superior overall survival.

In conclusion, our study has outlined unique epidemiological and genomic features of PLC-PHM MPC patients over the past two decades. Tailoring treatment approaches is vital for the improved management of this specific patient cohort. In the era of novel therapeutic modalities, the combination of lung cancer and hematologic malignancies represents a fortunate scenario. Further research is needed to elucidate the underlying pathogenic mechanisms contributing to the occurrence of MPC.

### Electronic supplementary material

Below is the link to the electronic supplementary material.

**Supplementary Material 1:** Integrated Analysis of Driver Gene Alterations, Treatment Modalities, and Regression Analysis in Cancer Research: Supplementary Tables

## Data Availability

No datasets were generated or analysed during the current study.
