# Supplementary material for: Emerging trends in the coexistence of primary lung Cancer and hematologic malignancy: a comprehensive analysis of clinicopathological features and genetic abnormalities
Source: Cancer Cell Int. 2024 Feb 24;24:84. doi: 10.1186/s12935-024-03264-x (PMC10893654; doi:10.1186/s12935-024-03264-x)
Supplement: Supplementary file 1 — Supplementary Material 1: Integrated Analysis of Driver Gene Alterations, Treatment Modalities, and Regression Analysis in Cancer Research: Supplementary Tables [file 12935_2024_3264_MOESM1_ESM.docx]

**Supplementary Table 1. Gene Status of the 10 Driver Genes in 38 MPC Lung Cancer Patient**

| Number | Gender | Pathology | EGFR | ALK | BRAF | ROS1 | MET | RET | KRAS | HER2 | PIK3C | TP53 |
| --- | --- | --- | --- | --- | --- | --- | --- | --- | --- | --- | --- | --- |
| 2 | Female | Adenocarcinoma | 1 | 0 | 0 | 0 | / | / | / | / | / | / |
| 5 | Male | Adenocarcinoma | 1 | / | / | / | / | / | / | / | / | / |
| 6 | Male | Adenocarcinoma | 1 | / | / | / | / | / | / | / | / | / |
| 7 | Female | Adenocarcinoma | 0 | 0 | 0 | 0 | 0 | 0 | 1 | 0 | 0 | 0 |
| 8 | Male | Adenosquamous | 1 | / | / | / | / | / | / | / | / | / |
| 10 | Female | Adenocarcinoma | 0 | 0 | / | / | / | / | / | / | / | / |
| 11 | Male | Squamous Cell | 0 | / | / | / | / | / | / | / | / | / |
| 17 | Female | Adenocarcinoma | 0 | 0 | / | 1 | 0 | / | / | / | / | / |
| 18 | Male | Adenocarcinoma | 1 | / | / | / | / | / | / | / | / | / |
| 19 | Male | Squamous Cell | 0 | 0 | / | 0 | / | / | / | / | / | / |
| 20 | Female | Adenocarcinoma | 1 | 0 | 0 | 0 | 0 | 0 | 0 | 0 | 0 | 0 |
| 21 | Male | Adenocarcinoma | 1 | 0 | 1 | 0 | 0 | 1 | 0 | 0 | 0 | 0 |
| 24 | Male | Squamous Cell | 0 | 1 | 0 | 0 | 0 | 0 | 0 | 0 | 0 | 1 |
| 25 | Male | Adenocarcinoma | 1 | / | / | / | / | / | / | / | / | / |
| 26 | Female | Adenocarcinoma | 1 | 0 | 0 | 0 | 0 | 0 | 0 | 1 | 0 | 1 |
| 29 | Female | Adenocarcinoma | 0 | 0 | 0 | 0 | 0 | 0 | 0 | 1 | 0 | / |
| 33 | Female | Adenocarcinoma | 1 | / | / | / | / | / | / | / | / | / |
| 34 | Male | Squamous Cell | 1 | 0 | 0 | 0 | 1 | 0 | 0 | 1 | 0 | 1 |
| 36 | Male | Adenocarcinoma | 1 | / | / | / | / | / | / | / | / | / |
| 37 | Female | Adenocarcinoma | 0 | 1 | / | / | / | / | 0 | / | / | / |
| 38 | Female | Adenocarcinoma | 1 | / | / | / | / | / | / | / | / | / |
| 39 | Male | Adenocarcinoma | 0 | / | / | / | / | 0 | / | 1 | / | / |
| 40 | Female | Adenocarcinoma | 1 | 0 | 0 | 0 | 0 | 1 | 0 | 0 | 0 | / |
| 41 | Female | Adenocarcinoma | 1 | 0 | 0 | 0 | 0 | 0 | 0 | 0 | 0 | / |
| 42 | Male | Adenocarcinoma | 1 | / | / | / | / | / | / | / | / | / |
| 45 | Male | Adenocarcinoma | 0 | 0 | 1 | 0 | 1 | 0 | 0 | 0 | / | / |
| 47 | Female | Adenocarcinoma | 0 | 0 | 0 | 0 | 0 | 1 | 0 | 0 | 0 | / |
| 48 | Male | Adenocarcinoma | 0 | 1 | / | / | / | / | / | / | / | / |
| 50 | Male | Adenocarcinoma | 0 | / | / | / | / | / | / | / | / | / |
| 51 | Male | Adenocarcinoma | 1 | / | / | / | / | / | / | / | / | / |
| 52 | Male | Adenocarcinoma | 0 | 0 | / | / | / | / | / | / | / | / |
| 53 | Male | Adenocarcinoma | 1 | 0 | 0 | 0 | / | / | / | / | / | / |
| 54 | Male | Adenocarcinoma | 1 | / | / | / | / | / | / | / | / | / |
| 65 | Male | Adenocarcinoma | 1 | 0 | 0 | 0 | / | / | / | / | / | / |
| 66 | Male | Squamous Cell | 0 | 0 | / | / | / | / | / | / | / | / |
| 74 | Female | Adenocarcinoma | 1 | 0 | 0 | 0 | / | / | / | / | / | / |
| 75 | Male | Small Cell | 0 | 0 | / | / | / | / | / | / | / | 1 |
| 78 | Female | Adenocarcinoma | 0 | 1 | / | / | / | / | / | / | / | / |
| Total |  |  | 21 | 4 | 2 | 1 | 2 | 3 | 2 | 3 | 0 | 4 |

Note: In the table, '1' represents that the gene was tested and found to be mutated, '0' indicates it was tested and found to be negative, and '/' denotes that it was not tested.

**Supplementary Table 2. Summary of Treatment Modalities for Different Hematologic Malignancies**

| Disease Category | Treatment Details | cases |
| --- | --- | --- |
| Mature B-cell Lymphoma (45 cases) | Surgery | 4 (8.89%) |
|  | Chemotherapy | 29 (64.44%) |
|  | Chemotherapy + Targeted therapy | 2 (4.44%) |
|  | Immunomodulatory therapy | 1 (2.22%) |
|  | No treatment | 2 (4.44%) |
|  | Treatment unclear | 7 (15.56%) |
| Acute Myeloid Leukemia (13 cases) | Chemotherapy | 8 (61.54%) |
|  | Targeted therapy | 1 (7.69%) |
|  | Symptomatic treatment | 3 (23.08%) |
|  | Treatment unclear | 1 (7.69%) |
| NK/T-cell Lymphoma (10 cases) | Surgery | 1 (10%) |
|  | Chemotherapy | 3 (30%) |
|  | Chemotherapy + Targeted therapy | 3 (30%) |
|  | Radiation therapy | 1 (10%) |
|  | Immunomodulatory therapy | 2 (20%) |
| Lymphoplasmacytic/Plasma Cell Tumors (9 cases) | Chemotherapy | 6 (66.67%) |
|  | Chemotherapy + Targeted therapy | 1 (11.11%) |
|  | Immunomodulatory therapy + Targeted therapy | 1 (11.11%) |
|  | Immunomodulatory therapy | 1 (11.11%) |
| Non-Hodgkin's Lymphoma (5 cases) | Combined Radiotherapy + Chemotherapy | 1 (20%) |
|  | Chemotherapy | 3 (60%) |
|  | Surgery + Radiotherapy + Chemotherapy | 1 (20%) |
| Myelodysplastic Syndrome (4 cases) | Chemotherapy | 1 (25%) |
|  | Targeted therapy | 2 (50%) |
|  | No treatment | 1 (25%) |
| Hodgkin's Lymphoma (3 cases) | Combined Radiotherapy + Chemotherapy | 1 (33.33%) |
|  | Chemotherapy + Immunotherapy | 1 (33.33%) |
|  | Chemotherapy | 1 (33.33%) |

**Supplementary Table 3. Univariate and Multivariate Regression Analysis**

| Characteristics | Total(N) | Univariate analysis | |  | Multivariate analysis | |
| --- | --- | --- | --- | --- | --- | --- |
|  |  | Hazard ratio  (95% CI) | P value |  | Hazard ratio  (95% CI) | P value |
| Gender | 85 |  |  |  |  |  |
| Male | 52 | Reference |  |  |  |  |
| Female | 33 | 0.938 (0.459 - 1.915) | 0.861 |  |  |  |
| Age | 85 |  |  |  |  |  |
| < 60 | 20 | Reference |  |  |  |  |
| ≥ 60 | 65 | 1.213 (0.523 - 2.817) | 0.653 |  |  |  |
| Smoking History | 85 |  |  |  |  |  |
| Yes | 28 | Reference |  |  |  |  |
| No | 57 | 1.007 (0.475 - 2.133) | 0.986 |  |  |  |
| Family History of Malignancies | 85 |  |  |  |  |  |
| Yes | 9 | Reference |  |  |  |  |
| No | 76 | 1.962 (0.595 - 6.472) | 0.268 |  |  |  |
| History of Comorbidities | 85 |  |  |  |  |  |
| Yes | 33 | Reference |  |  |  |  |
| No | 52 | 0.898 (0.445 - 1.813) | 0.764 |  |  |  |
| Order of Malignancies in Lung and Blood System Cancers | 85 |  |  |  |  |  |
| Blood Malignancy First | 48 | Reference |  |  |  |  |
| Lung Cancer First | 37 | 1.248 (0.628 - 2.483) | 0.527 |  |  |  |
| Synchronous MPC | 85 |  |  |  |  |  |
| Yes | 9 | Reference |  |  |  |  |
| No | 76 | 3.734 (0.508 - 27.436) | 0.195 |  |  |  |
| Time Interval for Lung Cancer and Blood System Malignancy in Metachronous Cancers | 85 |  |  |  |  |  |
| Over 10 Years | 9 | Reference |  |  | Reference |  |
| 5-10 Years | 17 | 0.776 (0.253 - 2.381) | 0.657 |  | 0.605 (0.158 - 2.320) | 0.464 |
| Within 5 Years | 50 | 0.276 (0.097 - 0.786) | 0.016 |  | 0.423 (0.131 - 1.361) | 0.149 |
| Lung Cancer Tissue Gene Mutation | 85 |  |  |  |  |  |
| Yes | 30 | Reference |  |  | Reference |  |
| No | 6 | 0.000 (0.000 - Inf) | 0.998 |  | 0.000 (0.000 - Inf) | 0.997 |
| Lung Cancer Surgical History | 85 |  |  |  |  |  |
| No | 25 | Reference |  |  | Reference |  |
| Yes | 57 | 0.393 (0.193 - 0.797) | 0.010 |  | 0.276 (0.083 - 0.918) | 0.036 |
| Lung Cancer Pathology | 85 |  |  |  |  |  |
| Squamous Cell Carcinoma | 13 | Reference |  |  | Reference |  |
| Adenocarcinoma | 59 | 0.617 (0.226 - 1.684) | 0.346 |  | 0.211 (0.049 - 0.916) | 0.038 |
| Small Cell Carcinoma | 5 | 3.010 (0.701 - 12.920) | 0.138 |  | 2.581 (0.501 - 13.297) | 0.257 |
| Lung Cancer Staging | 85 |  |  |  |  |  |
| Stage III-IV | 26 | Reference |  |  | Reference |  |
| Stage I-II | 42 | 0.417 (0.182 - 0.956) | 0.039 |  | 3.695 (0.823 - 16.589) | 0.088 |
| Lung Cancer PS Score | 85 |  |  |  |  |  |
| ≥2 | 4 | Reference |  |  | Reference |  |
| 0-1 | 64 | 0.110 (0.030 - 0.399) | < 0.001 |  | 0.079 (0.011 - 0.538) | 0.010 |
| Blood System Malignancy Type | 85 |  |  |  |  |  |
| Acute Myeloid Leukemia | 12 | Reference |  |  |  |  |
| Myeloproliferative Neoplasm | 4 | 0.555 (0.062 - 4.994) | 0.600 |  |  |  |
| Mature B-cell Lymphoma | 43 | 0.440 (0.139 - 1.397) | 0.164 |  |  |  |
| Hodgkin's Lymphoma | 3 | 1.461 (0.262 - 8.141) | 0.666 |  |  |  |
| Plasma Cell and Plasma Cell Tumor | 8 | 0.289 (0.051 - 1.632) | 0.160 |  |  |  |
| NK/T-cell Lymphoma | 10 | 1.443 (0.417 - 4.991) | 0.563 |  |  |  |
| Blood System Malignancy Genetic Alterations | 85 |  |  |  |  |  |
| None | 3 | Reference |  |  | Reference |  |
| Yes | 32 | 0.097 (0.024 - 0.387) | < 0.001 |  | 0.052 (0.010 - 0.279) | < 0.001 |
